# Comparable outcomes after McKeown and Ivor-Lewis minimally invasive esophagectomy with standardized two-field lymphadenectomy: a propensity score-matched study

**DOI:** 10.3389/fonc.2026.1879909

**Published:** 2026-08-24

**Authors:** Siyu Quan, Yun Li, Xianyun Cai, Fengqi Guo, Ya Wang, Hounai Xie

**Affiliations:** 1 Department of Thoracic Surgery, Shandong Provincial Hospital Affiliated to Shandong First Medical University, Jinan, China; 2 Medical Imaging Department, Shandong Provincial Hospital Affiliated to Shandong First Medical University, Jinan, China; 3 Department of Anesthesiology and Surgery, Shandong Provincial Hospital Affiliated to Shandong First Medical University, Jinan, China; 4 The 960th Hospital of the PLA Joint Logistics Support Force, Jinan, China

**Keywords:** esophageal cancer, Ivor-Lewis esophagectomy, McKeown esophagectomy, minimally invasive esophagectomy, overall survival, postoperative complications, propensity score matching

## Abstract

**Background:**

Minimally invasive esophagectomy (MIE) is widely used for esophageal cancer, but the optimal choice between McKeown and Ivor-Lewis MIE remains controversial. This study compared perioperative outcomes and 5-year overall survival (OS) between the two approaches under a standardized two-field lymphadenectomy framework.

**Methods:**

We retrospectively reviewed a cohort of 1,011 patients who underwent MIE for esophageal cancer between May 2017 and May 2020. To minimize selection bias, 1:1 propensity score matching (PSM) was performed using the following covariates: age, gender, body mass index(BMI), FEV1%, weight loss, albumin, smoking and drinking history, hypertension, diabetes, coronary heart disease, ASA score, histologic type, tumor location, TNM stage, and neoadjuvant therapy. The primary endpoint was 5-year OS, and the secondary endpoints were perioperative outcomes and postoperative complications.

**Results:**

After matching, 542 patients were included, with 271 patients in each group. In the matched cohort, overall postoperative complications were comparable between the McKeown and Ivor-Lewis groups (28.4% vs. 30.6%, P = 0.572), as were Clavien–Dindo grade III or higher complications (18.5% vs. 22.1%, P = 0.286), 30-day mortality (1.5% vs. 1.1%, P = 1.000), and 90-day mortality (2.6% vs. 3.3%, P = 0.612). Five-year OS was 43.5% and 36.2%, respectively, with no significant difference between groups after matching (log-rank P = 0.58). Multivariable Cox analysis showed that surgical approach was not independently associated with OS (Ivor-Lewis vs. McKeown: HR 0.93, 95% CI 0.74–1.16, P = 0.522), whereas prolonged postoperative hospital stay, tumor location, advanced TNM stage, and Clavien–Dindo grade III or higher complications were independently associated with OS.

**Conclusions:**

In this real-world propensity score-matched cohort, McKeown and Ivor-Lewis MIE achieved comparable perioperative safety and long-term OS under a standardized two-field lymphadenectomy framework. Long-term prognosis appeared to be more closely associated with tumor stage, tumor location, postoperative hospital stay, and severe postoperative complications than with the surgical approach itself.

## 1 Introduction

Esophageal cancer remains one of the most prevalent and lethal malignant tumors worldwide (1, 2).For patients with resectable esophageal cancer, esophagectomy combined with lymph node dissection remains a core component of radical treatment (3).Over the past decade, MIE has been widely adopted into routine clinical practice, as it offers comparable oncological radicality to open surgery while reducing surgical trauma, accelerating postoperative recovery, and improving short-term outcomes (4–6).

McKeown and Ivor-Lewis esophagectomy are the two most commonly used minimally invasive procedures for esophageal cancer (7).The main difference between them is the site of the anastomosis. McKeown esophagectomy uses a cervical anastomosis and is usually preferred when a wider upper mediastinal dissection is needed. By comparison, Ivor-Lewis esophagectomy uses an intrathoracic anastomosis and may have some technical advantages, such as less anastomotic tension (8, 9). However, the impact of these two approaches on postoperative complications and long-term survival remains controversial (7, 10–13).

Although a number of studies have compared McKeown and Ivor-Lewis esophagectomy, the current evidence still has several limitations (7, 10–13). First, some early studies were retrospective investigations involving small sample sizes, resulting in insufficient statistical power. Second, a portion of the literature dates back to a period when open esophagectomy remained the dominant approach—or to the transitional phase toward minimally invasive surgery—which likely introduced heterogeneity at the level of surgical practice. Third, while neoadjuvant therapy has emerged as a cornerstone of treatment for locally advanced esophageal cancer, its application in early studies was limited. Given these limitations, it remains difficult to ascertain the effect of surgical approach on postoperative complications and long-term survival.

Accordingly, we compared the perioperative outcomes and long-term survival of patients who underwent McKeown or Ivor-Lewis minimally invasive esophagectomy at a high-volume thoracic center. Propensity score matching was used to reduce baseline imbalance between the two groups. The study cohort included patients who received neoadjuvant therapy, reflecting real-world treatment practice during the study period (14, 15). All included patients underwent surgery under a standardized two-field lymphadenectomy framework. We aimed to compare perioperative outcomes, severe postoperative complications, and long-term OS between McKeown and Ivor-Lewis MIE, and to further explore prognostic factors associated with OS in a real-world propensity score-matched cohort.

## 2 Materials and methods

This study retrospectively analyzed the data of patients who underwent minimally invasive esophagectomy for esophageal cancer at the Department of Thoracic Surgery, Shandong Provincial Hospital Affiliated to Shandong First Medical University, between May 2017 and May 2020. All consecutive eligible patients who underwent totally minimally invasive McKeown or Ivor-Lewis esophagectomy during the study period were screened.

**Figure 1:**
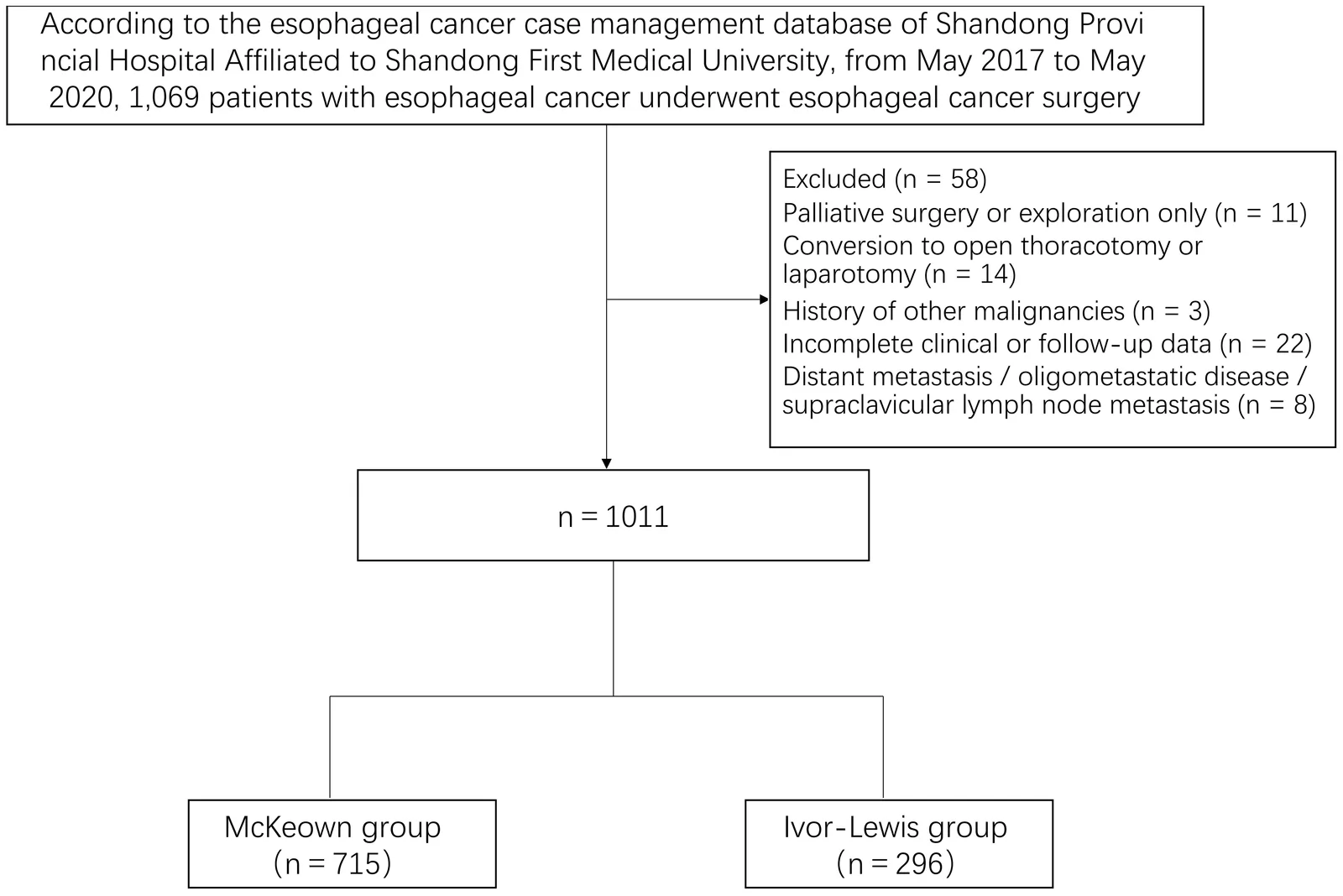
Flow diagram of patient selection.

The inclusion criteria were (1): histologically confirmed esophageal cancer (2); underwent totally minimally invasive McKeown or Ivor-Lewis esophagectomy (3); achieved R0 resection. The exclusion criteria were (1): palliative surgery or exploration only (2); conversion to open thoracotomy or laparotomy (3); history of other malignancies (4); incomplete clinical or follow-up data; (5) distant organ metastasis, oligometastatic disease, or supraclavicular lymph node metastasis at preoperative evaluation.(**Figure 1**).

Of note, the stage IV cases retained in the final cohort represented locally advanced, technically resectable disease with regional lymph node involvement rather than distant metastatic or palliative disease. These patients were considered suitable for radical esophagectomy after multidisciplinary assessment, and all underwent systematic lymphadenectomy with R0 resection.

The study was approved by the Ethics Committee of Shandong Provincial Hospital Affiliated to Shandong First Medical University (SWYX: NO.2025-748). Given the retrospective design of the study, the requirement for written informed consent from patients was waived by the ethics committee.

### 2.1 Preoperative workup and neoadjuvant therapy

Preoperative evaluation routinely included contrast-enhanced computed tomography (CT) of the chest and abdomen, cervical ultrasonography, and upper gastrointestinal endoscopy. Endoscopic ultrasonography was performed when necessary. Positron emission tomography-computed tomography (PET-CT) was additionally used in selected patients with locally advanced tumors or suspected distant metastasis. TNM stage was determined preoperatively according to the 8th edition of the AJCC staging system based on imaging, endoscopy, and other preoperative assessments.

Patients with locally advanced disease, defined as cT3–4 and/or clinically positive lymph nodes, were evaluated for neoadjuvant treatment after multidisciplinary team (MDT) discussion. When administered, neoadjuvant therapy was classified according to treatment modality based on preoperative medical records, including chemotherapy, chemoradiotherapy, radiotherapy alone, immunotherapy plus chemotherapy, and immunotherapy plus chemoradiotherapy. Surgery was usually scheduled 4–6 weeks after completion of neoadjuvant treatment.

### 2.2 Surgical techniques

In routine clinical practice, the choice between McKeown and Ivor-Lewis MIE was determined by tumor location, the required proximal resection margin, the feasibility of intrathoracic anastomosis, patient condition, and the operating surgeon’s judgment within a standardized surgical framework.All operations were performed by the same experienced thoracic surgical team after completion of the institutional learning curve for minimally invasive esophagectomy. In both groups, esophagectomy was performed under a standardized two-field lymphadenectomy framework, referring to thoracic mediastinal and abdominal lymphadenectomy.

#### 2.2.1 McKeown MIE

The operation consisted of three stages. With the patient in the left lateral decubitus position, the thoracic esophagus was mobilized and mediastinal lymph node dissection was performed using video-assisted thoracoscopic surgery (VATS). The patient was then repositioned supine for laparoscopic gastric mobilization and abdominal lymph node dissection. Finally, a left cervical incision was made, and the gastric conduit was brought up to the neck for anastomosis.

#### 2.2.2 Ivor-Lewis MIE

The procedure consisted of two stages. First, laparoscopic gastric mobilization and abdominal lymphadenectomy were performed in the supine position. The patient was then repositioned to the left lateral decubitus position for VATS esophageal mobilization and mediastinal lymphadenectomy. An intrathoracic esophagogastric anastomosis was performed using a stapler.

### 2.3 Endpoints and definitions

The primary endpoint was overall survival, defined as the interval from the date of surgery to all-cause death or the date of last follow-up. Perioperative outcomes included estimated blood loss, duration of surgery, harvested lymph node count, postoperative hospital stay, ICU admission, ICU stay duration, 30-day mortality, and 90-day mortality. ICU stay duration was analyzed only among patients admitted to the ICU, and 30-day and 90-day mortality were defined as all-cause death within 30 and 90 days after surgery, respectively.Postoperative complications were assessed during the index hospitalization or within 30 days after surgery.

Complications were defined according to the Esophagectomy Complications Consensus Group (ECCG) criteria where applicable (16). The complications of interest in this study included pneumonia, anastomotic leakage, chylothorax, atrial fibrillation, recurrent laryngeal nerve paralysis, and overall postoperative complications. The severity of postoperative complications was further assessed using the Clavien-Dindo classification, and severe postoperative complications were defined as Clavien-Dindo grade III or higher.

### 2.4 Follow-up

Patients were followed up through outpatient visits or telephone interviews. Follow-up was scheduled every 3 months during the first 2 years after surgery and every 6 months thereafter. Routine follow-up assessments included physical examination, serum tumor marker testing, and chest and abdominal CT.

### 2.5 Statistical analysis

Statistical analyses were performed using SPSS version 26.0. Continuous variables were expressed as mean ± standard deviation (SD) and compared using the independent samples t-test or Mann-Whitney U test. Categorical variables were presented as frequencies and compared using the Chi-square test or Fisher’s exact test.

To minimize selection bias, PSM was employed to balance baseline characteristics between the McKeown and Ivor-Lewis groups. Propensity scores were estimated using a logistic regression model including age, gender, body mass index, FEV1%, weight loss, albumin, smoking history, drinking history, hypertension, diabetes, coronary heart disease, American Society of Anesthesiologists score, histologic type, tumor location, TNM stage, and neoadjuvant therapy.Patients were matched 1:1 using nearest-neighbor matching without replacement, with a caliper width of 0.2 of the standard deviation of the logit of the propensity score. Covariate balance before and after matching was assessed using the standardized mean difference (SMD), and an SMD <0.1 was considered to indicate acceptable balance.

Overall survival was estimated using the Kaplan-Meier method and compared using the log-rank test. Median follow-up time was estimated using the reverse Kaplan-Meier method. Survival analyses were performed in both the unmatched cohort and the propensity score-matched cohort. Univariable and multivariable Cox proportional hazards regression models were used to identify factors associated with OS, and hazard ratios with 95% confidence intervals were calculated. Variables considered clinically relevant or showing potential association on univariable analysis were entered into the multivariable model. All statistical tests were two-sided, and P <0.05 was considered statistically significant.

## 3 Results

### 3.1 Patient characteristics and propensity score matching

A total of 1,011 patients were initially reviewed, including 715 in the McKeown group and 296 in the Ivor-Lewis group. Before matching, the two groups were generally comparable in age, gender, BMI, FEV1%, weight loss, albumin level, drinking history, hypertension, diabetes, coronary heart disease, and ASA score. However, significant between-group differences were observed in smoking history (P = 0.041), histologic type (P = 0.004), tumor location (P < 0.001), TNM stage (P = 0.034), and receipt of neoadjuvant therapy (P=0.004) (**Table 1**).

**Table 1:** Baseline characteristics of patients who underwent McKeown and Ivor-Lewis esophagectomy before and after propensity score matching.

Variable	Before matching				After matching			
	McKeown N = 715	Ivor-Lewis N = 296	p-value	Before SMD	McKeown N = 271	Ivor-Lewis N = 271	p-value	After SMD
Age	62.11 ± 7.68	62.55 ± 7.88	0.424	-0.056	61.95 ± 7.48	62.39 ± 7.89	0.506	-0.057
BMI	23.73 ± 3.45	23.49 ± 3.18	0.273	0.073	23.38 ± 3.35	23.44 ± 3.14	0.849	-0.016
FEV1%	99.74 ± 17.66	99.97 ± 17.19	0.848	-0.013	100.15 ± 16.43	100.34 ± 17.50	0.893	-0.012
Weight loss	1.27 ± 2.40	1.21 ± 2.46	0.684	0.028	1.12 ± 2.28	1.27 ± 2.54	0.476	-0.061
Albumin	40.99 ± 4.14	41.09 ± 3.93	0.734	-0.023	41.19 ± 4.39	41.13 ± 3.94	0.880	0.013
Gender			0.099	0.114			0.746	0.028
Male	603 (84.3%)	237 (80.1%)			219 (80.8%)	216 (79.7%)		
Female	112 (15.7%)	59 (19.9%)			52 (19.2%)	55 (20.3%)		
Smoker			0.041	0.141			0.860	0.015
0	313 (43.8%)	109 (36.8%)			105 (38.7%)	107 (39.5%)		
1	402 (56.2%)	187 (63.2%)			166 (61.3%)	164 (60.5%)		
Drinker			0.816	0.016			1.000	0.000
0	327 (45.7%)	133 (44.9%)			126 (46.5%)	126 (46.5%)		
1	388 (54.3%)	163 (55.1%)			145 (53.5%)	145 (53.5%)		
Hypertension			0.157	0.098			0.513	0.056
0	590 (82.5%)	233 (78.7%)			222 (81.9%)	216 (79.7%)		
1	125 (17.5%)	63 (21.3%)			49 (18.1%)	55 (20.3%)		
Diabetes			0.975	0.002			0.534	0.053
0	613 (85.7%)	254 (85.8%)			236 (87.1%)	231 (85.2%)		
1	102 (14.3%)	42 (14.2%)			35 (12.9%)	40 (14.8%)		
CHD			0.383	0.060			0.545	0.052
0	634 (88.7%)	268 (90.5%)			249 (91.9%)	245 (90.4%)		
1	81 (11.3%)	28 (9.5%)			22 (8.1%)	26 (9.6%)		
ASA score			0.694	0.062			0.430	0.068
II	539 (75.4%)	216 (73.0%)			206 (76.0%)	198 (73.1%)		
III	174 (24.3%)	80 (27.0%)			65 (24.0%)	73 (26.9%)		
I	1 (0.1%)	0 (0.0%)			0 (0.0%)	0 (0.0%)		
IV	1 (0.1%)	0 (0.0%)			0 (0.0%)	0 (0.0%)		
Pathology			0.004	0.226			1.000	0.033
Squamous carcinoma	644 (90.1%)	277 (93.6%)			255 (94.1%)	254 (93.7%)		
Others	27 (3.8%)	15 (5.1%)			13 (4.8%)	13 (4.8%)		
Adenomatous carcinoma	44 (6.2%)	4 (1.4%)			3 (1.1%)	4 (1.5%)		
Location			<0.001	0.934			0.896	0.011
Lower	408 (57.1%)	33 (11.1%)			34 (12.5%)	33 (12.2%)		
Middle	304 (42.5%)	263 (88.9%)			237 (87.5%)	238 (87.8%)		
Upper	3 (0.4%)	0 (0.0%)			0 (0.0%)	0 (0.0%)		
pT			0.226	0.160			0.998	-0.028
Tis	7 (1.0%)	3 (1.0%)			3 (1.1%)	3 (1.1%)		
1	115 (16.1%)	54 (18.2%)			49 (18.1%)	52 (19.2%)		
2	142 (19.9%)	64 (21.6%)			56 (20.7%)	56 (20.7%)		
3	395 (55.2%)	163 (55.1%)			151 (55.7%)	148 (54.6%)		
4	56 (7.8%)	12 (4.1%)			12 (4.4%)	12 (4.4%)		
pN			<0.001	0.221			0.082	-0.205
0	380 (53.1%)	166 (56.1%)			173 (63.8%)	156 (57.6%)		
1	158 (22.1%)	90 (30.4%)			56 (20.7%)	80 (29.5%)		
2	127 (17.8%)	30 (10.1%)			35 (12.9%)	26 (9.6%)		
3	50 (7.0%)	10 (3.4%)			7 (2.6%)	9 (3.3%)		
TNM Stage			0.034	0.205			0.891	0.078
Tis	7 (1.0%)	3 (1.0%)			3 (1.1%)	3 (1.1%)		
I	125 (17.5%)	52 (17.6%)			56 (20.7%)	51 (18.8%)		
II	244 (34.1%)	116 (39.2%)			109 (40.2%)	106 (39.1%)		
III	267 (37.3%)	112 (37.8%)			95 (35.1%)	99 (36.5%)		
IV	72 (10.1%)	13 (4.4%)			8 (3.0%)	12 (4.4%)		
Neoadjuvant therapy			0.004	0.274			0.821	0.088
No	541 (75.7%)	257 (86.8%)			234 (86.3%)	233 (86.0%)		
Immunotherapy plus chemotherapy	35 (4.9%)	11 (3.7%)			15 (5.5%)	10 (3.7%)		
Chemoradiotherapy	52 (7.3%)	10 (3.4%)			8 (3.0%)	10 (3.7%)		
Chemotherapy	61 (8.5%)	14 (4.7%)			12 (4.4%)	14 (5.2%)		
Immunotherapy plus chemoradiotherapy	17 (2.4%)	3 (1.0%)			2 (0.7%)	3 (1.1%)		
Radiotherapy alone	9 (1.3%)	1 (0.3%)			0 (0.0%)	1 (0.4%)		

CHD, coronary heart disease; ASA, American Society of Anesthesiologists.

After 1:1 propensity score matching, 542 patients remained in the matched cohort, including 271 patients in each group. Baseline characteristics were well balanced after matching. Covariate balance before and after matching is summarized in **Table 1** using standardized mean differences.

### 3.2 Perioperative outcomes

Perioperative outcomes in the unmatched and matched cohorts are summarized in **Table 2**. In the matched cohort, estimated blood loss was similar between the McKeown and Ivor-Lewis groups (175.98 ± 96.84 mL vs. 175.98 ± 103.85 mL, P = 1.000; **Table 2**). Duration of surgery also did not differ significantly between the two groups (273.25 ± 68.39 min vs. 269.89 ± 64.03 min, P = 0.555). The mean harvested lymph node count was 21.03 ± 9.97 in the McKeown group and 19.62 ± 6.68 in the Ivor-Lewis group; this difference did not reach statistical significance (P = 0.054). Postoperative hospital stay was also comparable between the two groups (12.15 ± 7.98 days vs. 12.21 ± 7.58 days, P = 0.930). No significant differences were observed in ICU admission (1.5% vs. 3.0%, P = 0.243), ICU stay duration among patients admitted to the ICU (1.25 ± 0.50 days vs. 2.25 ± 1.58 days, P = 0.136), 30-day mortality (1.5% vs. 1.1%, P = 1.000), or 90-day mortality (2.6% vs. 3.3%, P = 0.612) after matching.

**Table 2:** Perioperative and postoperative outcomes of patients undergoing McKeown and Ivor-Lewis esophagectomy before and after propensity score matching.

Variable	Before matching			After matching		
	McKeown N = 715	Ivor-Lewis N = 296	p-value	McKeown N = 271	Ivor-Lewis N = 271	p-value
EBL	168.48 ± 99.81	175.47 ± 103.78	0.324	175.98 ± 96.84	175.98 ± 103.85	1.000
DOS	269.59 ± 71.74	268.65 ± 63.51	0.836	273.25 ± 68.39	269.89 ± 64.03	0.555
LNC	20.69 ± 8.80	19.71 ± 6.53	0.052	21.03 ± 9.97	19.62 ± 6.68	0.054
ICU stay	1.62 ± 1.54 (n=16)	2.20 ± 1.48 (n=10)	0.354	1.25 ± 0.50 (n=4)	2.25 ± 1.58 (n=8)	0.136
Postoperative Hospital Stay	12.67 ± 22.43	12.29 ± 7.57	0.692	12.15 ± 7.98	12.21 ± 7.58	0.930
ICU admission			0.297			0.243
0	699 (97.8%)	286 (96.6%)		267 (98.5%)	263 (97.0%)	
1	16 (2.2%)	10 (3.4%)		4 (1.5%)	8 (3.0%)	
30-day mortality			0.766			1.000
0	705 (98.6%)	293 (99.0%)		267 (98.5%)	268 (98.9%)	
1	10 (1.4%)	3 (1.0%)		4 (1.5%)	3 (1.1%)	
90-day mortality			0.840			0.612
0	689 (96.4%)	286 (96.6%)		264 (97.4%)	262 (96.7%)	
1	26 (3.6%)	10 (3.4%)		7 (2.6%)	9 (3.3%)	
Pneumonia			0.598			0.386
0	578 (80.8%)	235 (79.4%)		222 (81.9%)	214 (79.0%)	
1	137 (19.2%)	61 (20.6%)		49 (18.1%)	57 (21.0%)	
AL			0.647			1.000
0	671 (93.8%)	280 (94.6%)		256 (94.5%)	256 (94.5%)	
1	44 (6.2%)	16 (5.4%)		15 (5.5%)	15 (5.5%)	
Chylothorax			0.768			0.285
0	704 (98.5%)	293 (99.0%)		265 (97.8%)	269 (99.3%)	
1	11 (1.5%)	3 (1.0%)		6 (2.2%)	2 (0.7%)	
AF			0.435			0.406
0	659 (92.2%)	277 (93.6%)		249 (91.9%)	254 (93.7%)	
1	56 (7.8%)	19 (6.4%)		22 (8.1%)	17 (6.3%)	
RLNP			0.407			0.523
0	669 (93.6%)	281 (94.9%)		261 (96.3%)	258 (95.2%)	
1	46 (6.4%)	15 (5.1%)		10 (3.7%)	13 (4.8%)	
Complications			0.821			0.572
0	490 (68.5%)	205 (69.3%)		194 (71.6%)	188 (69.4%)	
1	225 (31.5%)	91 (30.7%)		77 (28.4%)	83 (30.6%)	
Clavien–Dindo ≥ III complications			0.644			0.286
0	541 (75.7%)	228 (77.0%)		221 (81.5%)	211 (77.9%)	
1	174 (24.3%)	68 (23.0%)		50 (18.5%)	60 (22.1%)	

EBL, estimated blood loss; DOS, duration of surgery; LNC, Lymph Node Count; AL, anastomotic leakage; AF, atrial fibrillation; RLNP, recurrent laryngeal nerve paralysis.

No difference was observed in the overall postoperative complication rate between the two matched groups (28.4% vs. 30.6%, P = 0.572). Similarly, the incidences of pneumonia (18.1% vs. 21.0%, P = 0.386), anastomotic leakage (5.5% vs. 5.5%, P = 1.000), chylothorax (2.2% vs. 0.7%, P = 0.285), atrial fibrillation (8.1% vs. 6.3%, P = 0.406), and recurrent laryngeal nerve paralysis (3.7% vs. 4.8%, P = 0.523) were comparable between the two groups. The incidence of Clavien-Dindo grade III or higher complications was also similar between the McKeown and Ivor-Lewis groups (18.5% vs. 22.1%, P = 0.286).

### 3.3 Survival analysis

**Figure 2:**
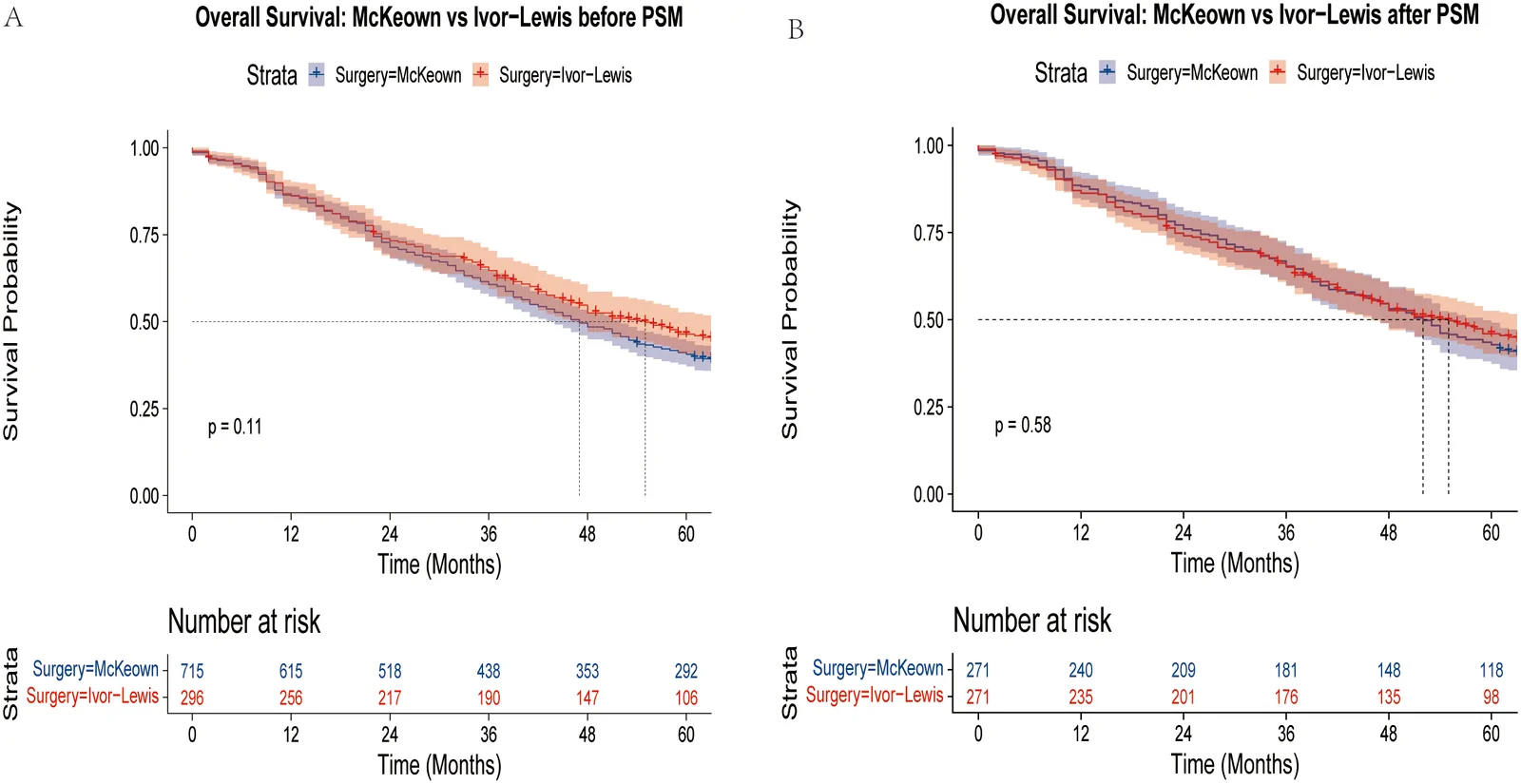
Kaplan–Meier survival curves comparing overall survival between the McKeown and Ivor-Lewis groups before and after propensity score matching. **(A)** Overall survival in the unmatched cohort before propensity score matching. **(B)** Overall survival in the matched cohort after propensity score matching.

Patients were enrolled between May 2017 and May 2020, and follow-up was continued until June 2025. The median follow-up time was 47.0 months (IQR, 22.0–73.0 months) in the unmatched cohort and 50.0 months (IQR, 24.0–72.8 months) in the matched cohort. Before matching, the 3-year OS rates were 61.3% and 64.2%, and the 5-year OS rates were 40.8% and 35.8% in the McKeown and Ivor-Lewis groups, respectively, with no statistically significant difference between groups (log-rank P = 0.11). After propensity score matching, the 3-year OS rates were 66.8% and 65.0%, and the 5-year OS rates were 43.5% and 36.2%, respectively, and the difference remained statistically non-significant (log-rank P = 0.58, **Figure 2**).

**Figure 3:**
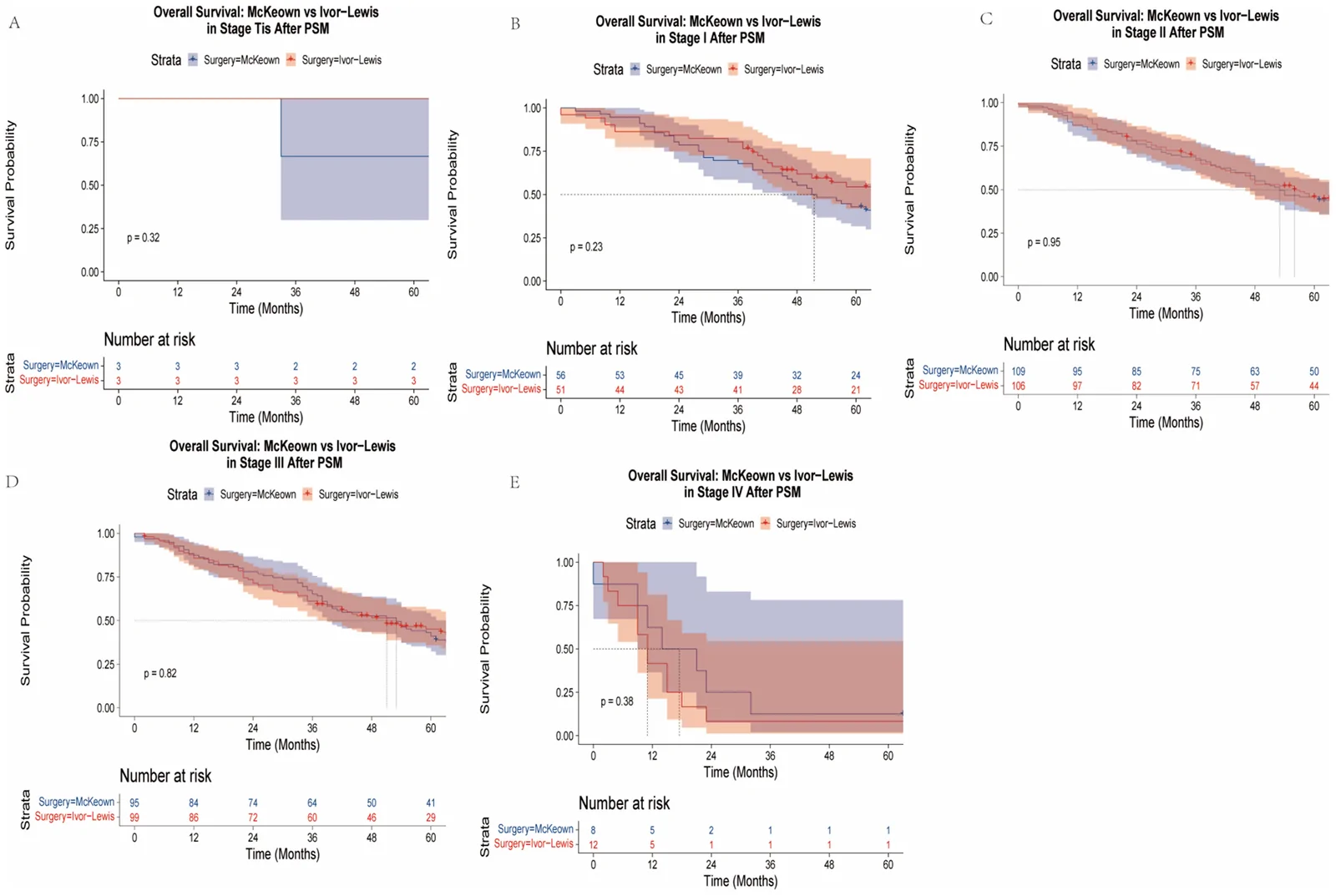
TNM stage-stratified Kaplan–Meier survival curves after propensity score matching. Overall survival was compared between the McKeown and Ivor-Lewis groups within each TNM stage subgroup in the matched cohort. **(A)** Tis stage; **(B)** stage I; **(C)** stage II; **(D)** stage III; and **(E)** stage IV.

To further assess whether the comparison between the two surgical approaches was influenced by TNM stage, we performed TNM stage-stratified Kaplan–Meier analyses in the matched cohort. Within each TNM stage subgroup, no statistically significant difference in OS was observed between the McKeown and Ivor-Lewis groups (Tis: log-rank P = 0.32; stage I: P = 0.23; stage II: P = 0.95; stage III: P = 0.82; stage IV: P = 0.38; **Figure 3**).

### 3.4 Prognostic factors for overall survival

**Figure 4:**
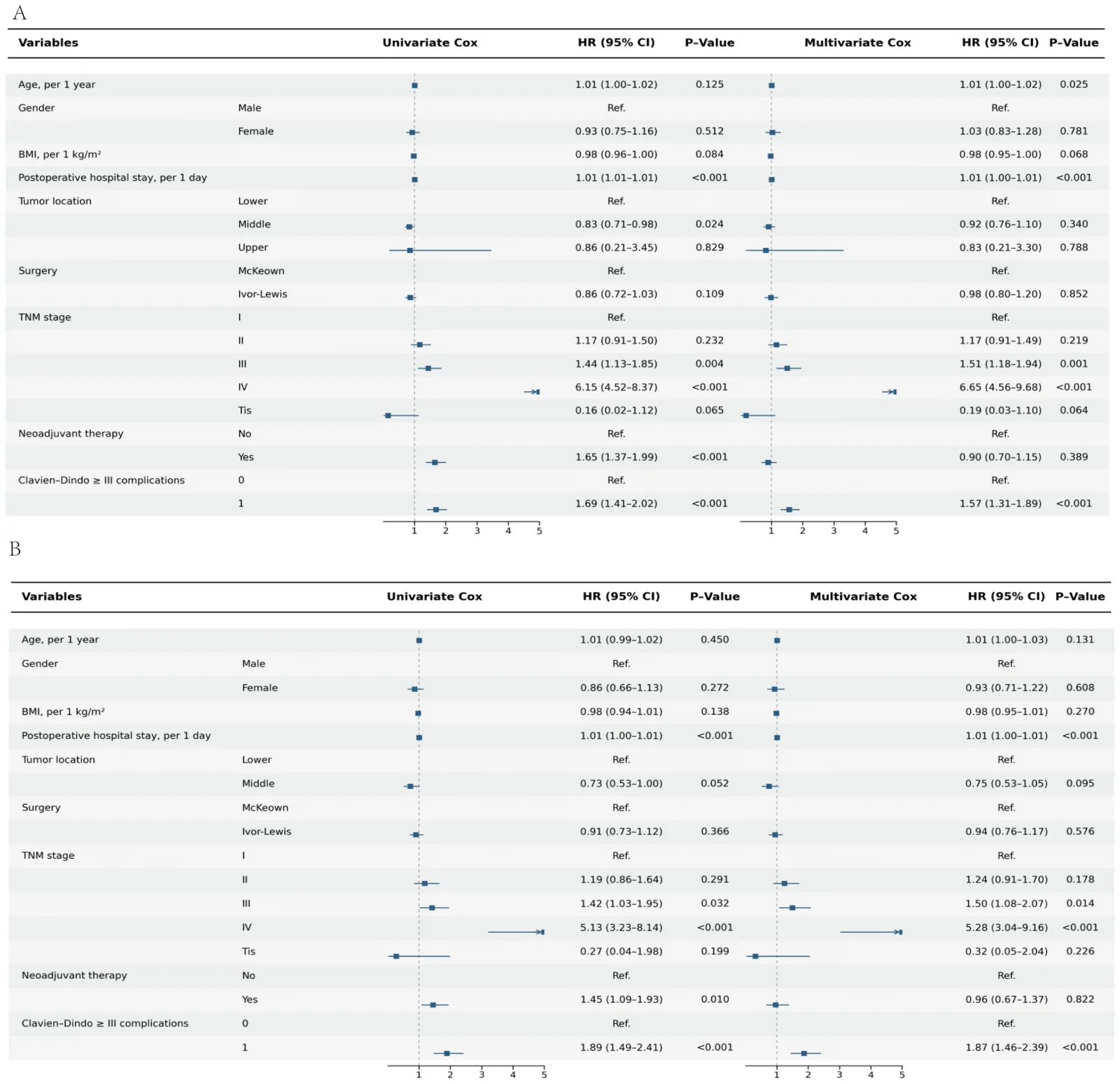
Univariate and multivariate Cox regression analyses for overall survival before and after propensity score matching. **(A)** Cox regression analysis before propensity score matching. **(B)** Cox regression analysis after propensity score matching. In each panel, the left section presents univariate Cox regression results, whereas the right section presents multivariate Cox regression results. Dots represent hazard ratios, and horizontal lines represent 95% confidence intervals. The vertical reference line indicates a hazard ratio of 1.0. A hazard ratio greater than 1.0 indicates an increased risk of death, whereas a hazard ratio less than 1.0 indicates a decreased risk.

Univariable and multivariable Cox proportional hazards regression analyses were performed in both the unmatched and propensity score-matched cohorts to identify factors associated with OS (**Figure 4**). In the unmatched cohort, surgical approach was not independently associated with OS on multivariable analysis (Ivor-Lewis vs. McKeown: HR 0.98, 95% CI 0.80–1.20, P = 0.852). Age as a continuous variable, per 1-year increase (HR 1.01, 95% CI 1.00–1.02, P = 0.025), longer postoperative hospital stay (HR 1.01, 95% CI 1.00–1.01, P < 0.001), stage III disease (HR 1.51, 95% CI 1.18–1.94, P = 0.001), stage IV disease (HR 6.65, 95% CI 4.56–9.68, P < 0.001), and Clavien-Dindo grade III or higher complications (HR 1.57, 95% CI 1.31–1.89, P < 0.001) were independently associated with poorer OS.

In the matched cohort, surgical approach was not independently associated with OS on multivariable analysis (Ivor-Lewis vs. McKeown: HR 0.93, 95% CI 0.74–1.16, P = 0.522). Postoperative hospital stay remained independently associated with OS (HR 1.03, 95% CI 1.02–1.05, P < 0.001). Compared with lower thoracic tumors, middle thoracic tumors were associated with a lower hazard of death after adjustment (HR 0.68, 95% CI 0.49–0.95, P = 0.022). Advanced TNM stage was also independently associated with poorer OS, particularly stage III disease (HR 1.40, 95% CI 1.01–1.95, P = 0.044) and stage IV disease (HR 6.80, 95% CI 3.50–13.22, P < 0.001). Clavien-Dindo grade III or higher complications remained an independent adverse prognostic factor in the matched cohort (HR 1.61, 95% CI 1.22–2.12, P < 0.001; **Figure 4**).

## 4 Discussion

The optimal minimally invasive approach for esophageal cancer remains controversial. In this study, we compared McKeown and Ivor-Lewis minimally invasive esophagectomy in a high-volume center using a standardized two-field lymphadenectomy procedure. After propensity score matching, the two procedures showed comparable perioperative outcomes, including operative time, estimated blood loss, lymph node count, postoperative hospital stay, ICU admission, ICU stay duration, 30-day mortality, 90-day mortality, overall postoperative complications, and Clavien-Dindo grade III or higher complications. More importantly, no significant difference was found in 5-year overall survival after matching. These results suggest that when surgical procedures and perioperative management are relatively standardized, the choice of approach may have limited impact on short-term outcomes and long-term survival.

Previous studies have suggested that the McKeown approach is associated with a higher incidence of anastomotic leakage and recurrent laryngeal nerve injury (7–9, 11, 13). This is usually explained by the need for cervical anastomosis and wider dissection in the upper mediastinum. Compared with intrathoracic anastomosis, cervical anastomosis requires the gastric conduit to be brought up to the neck, which may increase anastomotic tension and make the distal conduit blood supply more vulnerable (8, 9). In addition, more extensive dissection in the upper mediastinum may increase the chance of traction or thermal injury to the recurrent laryngeal nerve.

However, such differences were not observed in this study. This finding should be interpreted in the context of surgical standardization and institutional experience. The absence of significant between-group differences in these complications may be partly explained by the fact that both procedures were performed by an experienced surgical team using standardized techniques. In the present study, all operations were performed by an experienced high-volume surgical team after completion of the learning curve for minimally invasive esophagectomy. During gastric conduit construction, attention was paid to preserving conduit blood supply and ensuring adequate conduit length to reduce anastomotic tension. Cervical anastomoses were reinforced when appropriate. In addition, the magnified thoracoscopic view may facilitate clearer identification of the recurrent laryngeal nerve and adjacent structures during upper mediastinal lymphadenectomy, allowing careful dissection and reducing traction or thermal injury. Previous studies have shown that surgical performance, learning curve status, and procedural proficiency can substantially influence postoperative morbidity and long-term outcomes after esophagectomy (17–19). Therefore, the comparable rates of anastomotic leakage and recurrent laryngeal nerve paralysis observed in this study may be partly attributable to standardized operative procedures, careful anastomotic technique, recurrent laryngeal nerve protection, and accumulated institutional experience.

We also found no significant difference in long-term survival between the two groups. This suggests that long-term prognosis after esophagectomy may depend more on tumor stage and perioperative recovery than on the surgical procedure itself (20–22). Several factors may explain this finding. First, both McKeown and Ivor-Lewis MIE were performed under the same standardized two-field lymphadenectomy framework, and no significant differences were observed in harvested lymph node count, estimated blood loss, or duration of surgery after matching. Previous studies have suggested that when lymph node assessment, radical resection, and other oncological parameters are comparable, long-term survival may not differ significantly between Ivor-Lewis and McKeown esophagectomy (10). Therefore, the comparable extent of lymphadenectomy and surgical radicality achieved in the present study may have reduced the potential influence of surgical approach on OS. Second, neoadjuvant therapy may improve tumor control and long-term survival in selected patients (23), and its use in part of this cohort may have further attenuated the relative contribution of surgical approach to OS. Third, perioperative recovery and severe postoperative complications may have an effect on long-term outcomes (20). In this study, postoperative hospital stay, ICU admission, ICU stay duration, 30-day mortality, 90-day mortality, and Clavien-Dindo grade III or higher complications were comparable between the two matched groups. This may partly explain the similar long-term OS observed after matching. Finally, all procedures were performed by an experienced high-volume surgical team using standardized operative techniques after completion of the learning curve for minimally invasive esophagectomy. Surgical performance, learning curve status, and procedural proficiency have been reported to influence postoperative morbidity and long-term outcomes after esophagectomy (17, 25, 26).

Multivariable Cox analysis showed that surgical approach was not independently associated with OS in either the unmatched or matched cohort. By contrast, postoperative hospital stay, advanced TNM stage, tumor location, and Clavien-Dindo grade III or higher complications were independently associated with OS in the matched cohort. Taken together, these findings suggest that long-term survival after esophagectomy may be influenced more by tumor burden, tumor location, and postoperative recovery than by the choice of surgical approach itself. TNM stage reflects the extent of disease, and more advanced stage is usually associated with poorer prognosis (24). Severe postoperative complications may also adversely affect long-term outcomes by delaying recovery, impairing nutritional and functional status, postponing subsequent treatment, and increasing systemic inflammatory stress (25, 26). Therefore, accurate preoperative staging, standardized surgical procedures, and the prevention and management of severe postoperative complications remain important for improving long-term outcomes.

Among these prognostic factors, tumor location deserves further consideration. Tumor location is not only an anatomical descriptor but may also reflect differences in lymphatic drainage, tumor biology, surgical indication, and the extent of lymphadenectomy required. In the 8th edition of the AJCC staging system, tumor location is considered in the staging framework (24). In clinical practice, tumor location also strongly influences the choice between McKeown and Ivor-Lewis procedures. Tumors requiring a higher proximal margin or more extensive upper mediastinal lymphadenectomy are more likely to be treated with McKeown MIE, whereas Ivor-Lewis MIE is more commonly selected when intrathoracic anastomosis is considered oncologically feasible. Therefore, tumor location may act as both a prognostic factor and a determinant of surgical approach.

This study is subject to certain limitations. First, this was a retrospective single-center study, and residual confounding could not be completely eliminated despite propensity score matching. Second, this study focused on OS and did not include recurrence-free survival, detailed recurrence patterns, or post-recurrence treatment because recurrence-related data were not systematically and uniformly available, particularly for patients who received follow-up or subsequent treatment at local hospitals. Third, although neoadjuvant therapy was categorized by treatment modality, detailed drug regimens, treatment cycles, RECIST-based response assessments, and postoperative adjuvant therapy information were incompletely recorded in some patients and therefore could not be fully incorporated into the survival analysis. Fourth, although total harvested lymph node count was compared between the two surgical approaches, station-specific or compartment-specific lymph node counts were not systematically recorded in this retrospective database. Therefore, we could not quantitatively compare the exact nodal stations or lymphadenectomy compartments dissected between the McKeown and Ivor-Lewis procedures. Finally, upper thoracic tumors were uncommon in this real-world cohort and were absent from the matched cohort after propensity score matching. Therefore, the present findings should be interpreted mainly in the context of middle-to-lower thoracic esophageal cancer and should not be directly generalized to upper thoracic tumors. Further multicenter studies are needed to validate these results.

## 5 Conclusion

In this propensity score-matched study, no significant differences in perioperative outcomes or 5-year overall survival were observed between McKeown and Ivor-Lewis minimally invasive esophagectomy when both procedures were performed under a standardized two-field lymphadenectomy framework. Surgical approach was not independently associated with OS, whereas TNM stage, tumor location, postoperative hospital stay, and Clavien–Dindo grade III or higher complications were associated with long-term prognosis. These findings suggest that, in predominantly middle-to-lower thoracic esophageal cancer, surgical approach may be selected on an individualized basis, while greater emphasis should be placed on staging accuracy, perioperative recovery, and prevention of severe postoperative complications.

## Data Availability

The original contributions presented in the study are included in the article/supplementary material. Further inquiries can be directed to the corresponding author.
